# The effect of 3‐year parental smoking on asthma status of their children

**DOI:** 10.1111/crj.13492

**Published:** 2022-05-11

**Authors:** Mostafa Boskabady, Ali A. Hajizadeh, Hamid Ahanchian, Arghavan Memarzia, Maryam Jafarnezhad, Armin Golafshani, Mohammad H. Boskabady

**Affiliations:** ^1^ Applied Biomedical Research Center Mashhad University of Medical Sciences Mashhad Iran; ^2^ Department of Physiology, Faculty of Medicine Mashhad University of Medical Sciences Mashhad Iran; ^3^ Department of Pediatric Allergy‐Immunology Mashhad University of Medical Science Mashhad Iran; ^4^ Student Research Committee Mashhad University of Medical Sciences Mashhad Iran; ^5^ Department of Anesthesia Mashhad Medical Sciences Branch Islamic Azad University Mashhad Iran; ^6^ Clinical Supervisor of Hasheminejad Hospital Mashhad University of Medical Scince Mashad Iran

**Keywords:** asthma, children, pulmonary function tests, respiratory symptoms, smoking

## Abstract

**Objective:**

Whilst the prevalence and severity of asthma influenced by environmental factors, the effect of parental smoking on asthma status of their children was examined.

**Patients and Methods:**

Ninety asthmatic children, 32 with smoker and 58 with non‐smoker parents (baseline age, 8.5 ± 3.5 and 8.2 ± 3.3 respectively) were studies in two sessions 3 years apart by evaluating respiratory symptoms (RS) prevalence and severity, various drugs used, and pulmonary function tests (PFT) including forced vital capacity; forced volume in the first second, peak expiratory flow; and maximum expiratory low at 75, 50 and 25% of vital capacity (FVC, FEV1, PEF, MEF75, MEF50 and MEF25, respectively).

**Results:**

The prevalence and severity of all RS were significantly increased in asthmatic children with smoking parents after 3 years except prevalence and severity of night wheeze and the prevalence of chest wheeze (*p* < 0.05 to *p* < 0.001), but the PFT values were non‐significantly reduced. In asthmatic children with non‐smoking parents, the prevalence and severity of RS were decreased after 3 years, which was significant for night and chest wheeze for prevalence and night cough and chest wheeze for severity (all, *p* < 0.05), and the PFT values were increased, which were statistically significant for FVC, FEV1, MEF50 and MEF25 (*p* < 0.05 to *p* < 0.01). Drugs used by the group with smoking parents were increased and were significantly higher than their reduction in the groups with non‐smoking parents at the end of the study (*p* < 0.05 for fluticasone propionate 125/salmeterol and budesonide160/formoterol).

**Conclusion:**

Long‐term parental smoking increased prevalence and severity of RS and drug used but decreased PFT values of their asthmatic children.

## Graphical abstract

The effect of 3‐year parental smoking on asthma status of their children was examined. The prevalence and severity of respiratory were significantly higher, but the PFT values were lower in asthmatic children with smoking parents compared to those of non‐smoking parents at the end of the study. Long‐term parental smoking affects their asthmatic children status.

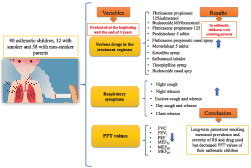

**List of Abbreviations**
CW: chest wheeze
DCW: day cough and wheeze
ECW: excises cough and wheeze
FEV_1_
: forced expiratory volume in one second
FVC: forced vital capacity
MEF_75_, MEF_50_ and MEF_25_
: maximal expiratory flow at 75%, 50% and 25% of the FVC
MEF_75_, MEF_50_ and MEF_25_
: maximal expiratory flow at 75%, 50% and 25% of the FVC
NC: night dough
NSP: non‐smoker parents
NW: night wheeze
PEF: peak expiratory flow
PFT: pulmonary function tests
RS: respiratory symptoms
SP: smoker parents

## 1 INTRODUCTION

Asthma is a common and widespread disease in the world that is associated with inflammation of respiratory tract^1^, ^2^ and hyperactive responsiveness, obstruction and remodelling of the airways.^3^ More than 300 million patients are suffering from asthma worldwide,^4^ which is markedly more prevalent in children and women.^5^ Environmental factors such as smoking, air pollution and allergens, lung infections, rhinitis and obesity affect asthma prevalence and severity in children and adults.^5^ The common symptoms of asthma are wheeze, chest tightness and cough,^5^ but improving of life style and using controlled drugs can decrease the symptoms of asthma.^6^

Allergens are the most important factors that implicated the asthma symptoms, and decreasing the allergen exposure can improve the asthma symptoms and prevalence of asthma in children in school ages.^7^, ^8^ It was shown that the risk of prevalent asthma in childhood increased about 40%, by smoking of either parent and postnatal maternal smoking increased the incidence of asthma before the age of 6 by ∼30%, and during school‐age years by 13%. Exposure to smoking increased the risk of incidence of asthma in childhood by 33%,^9^ and a significant association between paternal smoking and the risk of asthma in children was also reported.^8^ Smoking increased the airway mucosal permeability, the inflammatory cytokines, and the thickness and responsiveness of the airways but suppressed histone deacetylase activity.^10^ Asthmatic children exposed to multiple household smokers face an increased risk for respiratory illness‐related absences from school.^10^ Pulmonary function tests (PFT) were also decreased in children exposed to passive smoking.^11^, ^12^, ^13^

In this study, the effect of 3‐year parental smoking on respiratory symptoms (RS) using various drugs and PFT in their asthmatic children were examined.

## 2 MATERIAL AND METHODS

### 2.1 Subjects

Ninety asthmatic children, 32 with smoker parents (SP) and 58 with non‐smoker parents (NSP) with mild to moderate disease,^11^ were studied from an asthma clinic in Mashhad University of Medical Sciences with inclusion and exclusion criteria as previously described.^14^ Asthma severity of studied asthmatic children was assessed according criteria of the Global Strategy for Asthma Management and Prevention.^4^ The parent's smoking status did not change during the 3‐year study period. The patients were studied in two sessions 3 years apart during July–September 1998 and 2000 whilst they were under continues management and receiving their regular medications during the study period. The Ethical Committee of Mashhad University of Medical Sciences approved the study (Code: 897000), and the parents of all children were given informed consent. RS, PFT and prescribed drugs for the management of the disease were evaluated in the beginning and at the end of the study.

### 2.2 Drugs used by asthmatic children

Drugs in the treatment regimen of the asthmatic children included fluticasone propionate/salmeterol inhaler, budesonide/formoterol inhaler, fluticasone propionate inhaler, fluticasone propionate nasal spray, budesonide nasal spray, montelukast tablet, ketotifen syrup, salbutamol inhaler and theophylline syrup which were evaluated at the beginning and the end of the study.

### 2.3 Respiratory symptoms

The prevalence and severity of RS including wheezing, tightness and cough were evaluated using a Farsi questionnaire which was designed and used in the similar studies.^15^, ^16^, ^17^, ^18^ The chest wheeze was scored by a physician from 0 to 3 as follows: no wheezing = 0, hardly heard wheezing = 1, moderate wheezing = 2 and loud wheezing = 3 (Table 1).

**TABLE 1:** The criteria for respiratory symptom severity score

Symptom	Frequency	Score
Night wheeze	None	0
	Rarely (less than once a week)	1
	Occasionally (2–3/week)	2
	Most nights	3
Night cough	None	0
	Rarely (less than once a week)	1
	Occasionally (2–3/week)	2
	Most nights	3
Excises cough and wheeze	None	0
	During mild exercise (walking)	1
	During heavy exercise	2
	At rest	3
Day cough and wheeze	None	0
	Rarely (less than once a week)	1
	Occasionally (2–3/week)	2
	Most days	3
Chest wheeze	None	0
	Hardly hearing with statoscope	1
	Relative easily hearing with statoscope	2
	Loudly hearing with statoscope	3
Total score		14

### 2.4 Pulmonary function tests

Pulmonary function tests (PFT) were measured as previously described [14]. Various measured PFT values were included: forced vital capacity, forced expiratory volume in 1 s, peak expiratory flow, and maximal expiratory flow at 75%, 50% and 25% of the vital capacity (FVC, FEV_1_, PEF, MEF_75_, MEF_50_ and MEF_25_, respectively). The spirometry was performed in the studied children by a trained technician who was blind to smoking status of the children.

### 2.5 Statistics

Using the PPS sampling method, a minimum of 32 subjects in each group with an α error of 0.05 and a power of 80% was calculated. In this study, 58 asthmatic children with NSP and 32 children with SP were studied. The data of age, RS severity and PFT values as well as changes in RS severity and PFT values were expressed as mean ± SD.

The absolute values of all variables (RS, PFT values and used drugs for management of the disease) were compared between the beginning and the end of the study in each group as well as between two groups. In addition, the percentage changes in RS severity and PFT values during 3‐year study period were also calculated and compared between two groups.

The percentage changes in RS severity and PFT values during the study period were calculated using the following equation:

$$\frac{\text{Values}\;\mathrm{at}\;\text{the}\;\mathrm{end}\;\text{of the study}-\text{Values in the beginning of the study}}{\text{Values in the beginning of the study}}\;\times 100$$

Data normality test was done by Kolmogorov–Smirnov test. The data with normal distribution were compared between the baseline and the end of 3 years and also between two groups using parametric test (paired/unpaired *t* test) and for non‐normal distributed data; the non‐parametric (Wilcoxon/Mann–Whitney) tests were used. Differences in the data of RS prevalence between two groups were tested by Chi‐squared analysis (2 × 2 contingency tables). A *p* value of 0.05 was the criterion for statistical significance. All analyses were performed using SPSS software (version 11.5, SPSS Inc. USA).

## 3 RESULTS

### 3.1 General characteristics of asthmatic children in two groups

There was no significant difference in age, height and asthma severity between two groups (Table 2).

**TABLE 2:** General characteristics of studied asthmatic children in two groups

Character		Non‐smokers	Smokers
Number		58	32
Male		34 (58.6%)	19 (59.4%)
Female		24 (41.4%)	13 (40.6%)
Age	Baseline	8.19 ± 3.28	8.45 ± 3.52
	End	11.15 ± 3.27	11.45 ± 3.52
Height		137.33 ± 16.47	135.91 ± 15.69
Asthma severity	Mild	19 (32.76%)	12 (37.50%)
	Moderate	35 (60.34%)	18 (56.25%)
	Severe	4 (7.00%)	2 (6.25%)

### 3.2 The effect of parental smoking on drugs used by asthmatic children

Various types of drugs used by two groups of asthmatic children were not significantly different in the beginning of the study, but all drugs used by asthmatic children with NSP were non‐significantly decreased at the end of the study. Different types of drugs used by asthmatic children with SP were non‐significantly higher than those of NSP at the end of the study period (Table 3).

**TABLE 3:** Various drugs in the treatment regimen of asthmatic children of two groups at the beginning and at the end of the study

Drugs	Non‐smokers					Smokers					*p* value End vs NS
	Beginning		End		*p* value vs Beg	Beginning		End		*p* value vs Beg	
	No	%	No	%		No	%	No	%		
Fluticasone propionate 125/salmeterol	26	44.82%	18	31.03%	NS	14	43.75%	15	46.87%	NS	NS
Budesonide160/formoterol	16	27.58%	11	20.70%	NS	9	28.12%	11	34.37%	NS	NS
Fluticasone propionate 125	5	8.62%	4	6.89%	NS	3	9.37%	3	9.37%	NS	NS
Prednisolone 5 tablet	4	7.00%	2	3.44%	NS	2	6.25%	2	6.25%	NS	NS
Fluticasone propionate nasal spray	12	20.70%	8	13.79%	NS	7	21.87%	7	21.87%	NS	NS
Budesonide nasal spry	6	10.34%	4	6.89%	NS	3	9.37%	3	9.37%	NS	NS
Montelukast 5 tablet	10	17.24%	7	12.07%	NS	6	18.75%	6	18.75%	NS	NS
Ketotifen syrup	14	24.13%	14	24.13%	NS	8	25%	8	25%	NS	NS
Salbutamol inhaler	10	17.24%	6	10.34%	NS	6	18.75%	6	18.75%	NS	NS
Theophylline syrup	12	20.70%	8	13.79	NS	7	21.87%	6	18.75%	NS	NS

Beg: beginning, NS: non‐smokers, vs Beg: statistical comparison between the beginning and the end of 3 years of the study period, End vs NS: the statistical comparison between asthmatic children with smoker parents with those of non‐smokers at the end of the study period.

### 3.3 The effect of parental smoking on RS

**FIGURE 1:**
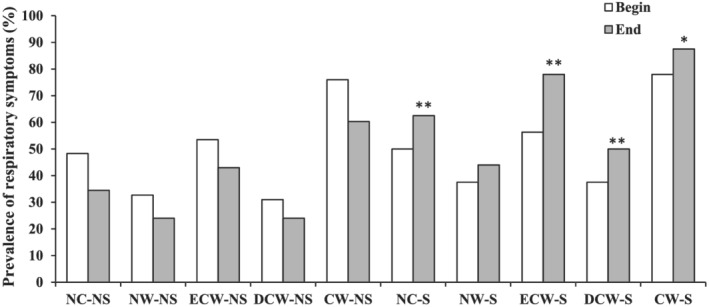
The prevalence of respiratory symptoms in asthmatic children with non‐smoker (NS) (*n* = 58) and smoker (S) parents (*n* = 32) at the beginning (big) and at the end (end) of the study period. Chi‐square was used for comparison between the results of two groups. *p < 0.05 and **p < 0.01 compared to non‐smoker groupNC: night dough, NW: night wheeze, ECW: excises cough and wheeze, DCW: day cough and wheeze, CW: chest wheeze, NS: asthmatic children with non‐smoker parents, S: asthmatic children with smoker parents.

The prevalence of RS between two groups at the beginning of the study was not significantly different. The prevalence of all RS in the asthmatic children with NSP was non‐significantly decreased, but in the group with SP was increased after 3 years (Table 4). All RS in asthmatic children with SP were significantly higher than those of NSP at the end of the study except night wheeze (*p* < 0.05 to *p* < 0.01) (Figure 1).

**TABLE 4:** The severity of respiratory symptoms in asthmatic children in two groups at the beginning and at the end of the study

Symptoms	Non‐smokers			Smokers			*p* value	
	Beg	End	*p* value vs Beg	Beg	End	*p* value vs Beg	Beg vs NS	End vs NS
Night cough	1.36 ± 0.18	0.97 ± 0.17	NS	1.31 ± 0.24	1.59 ± 0.23	NS	NS	<0.05
Night wheeze	0.97 ± 0.18	0.70 ± 0.16	NS	0.93 ± 0.23	1.06 ± 0.23	NS	NS	NS
Excises cough and wheeze	1.52 ± 0.17	1.18 ± 0.18	NS	1.55 ± 0.24	1.87 ± 0.21	NS	NS	<0.01
Day cough and wheeze	0.84 ± 0.16	0.63 ± 0.14	NS	0.89 ± 0.20	1.03 ± 0.18	NS	NS	<0.05
Chest wheeze	1.86 ± 0.13	1.43 ± 0.16	<0.05	1.89 ± 0.20	2.06 ± 0.18	NS	NS	<0.01

Beg: beginning, NS: non‐smokers, vs Beg vs NS Beg: statistical comparison between the beginning and the end of 3 years of the study period, Beg vs NS and End vs NS: statistical comparison between asthmatic children with smoker parents and those on non‐smokers at the beginning and the end of the study period, respectively.

**FIGURE 2:**
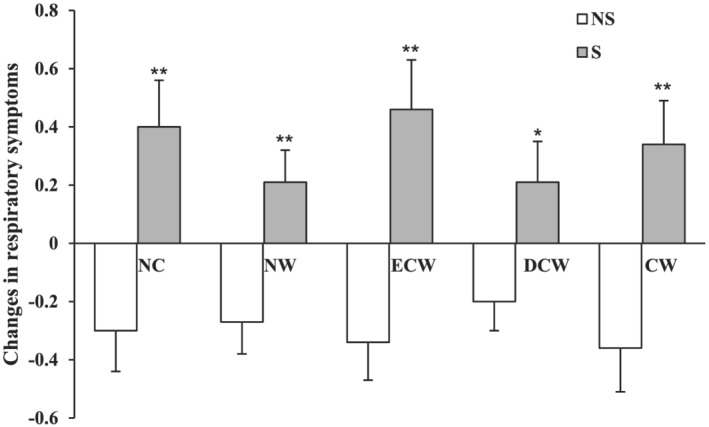
The absolute changes in respiratory symptoms severity during 3‐year study period in asthmatic children with non‐smoker (NS) (*n* = 58) and smoker (S) parents (*n* = 32). Paired *t* test was used for comparison between the results of two groups. ***p < 0.01 compared to non‐smoking groupNC: night dough, NW: night wheeze, ECW: excises cough and wheeze, DCW: day cough and wheeze, CW: chest wheeze. NS: asthmatic children with non‐smoker parents, S: asthmatic children with smoker parents.

The severity of all RS in NSP group was decreased at the end of the study, which was non‐statistically significant for CW (*p* < 0.01) (Table 4). However, the score of the all RS was non‐significantly increased in children with SP after 3 years (Table 4). The severity of all RS in SP group at the end of 3‐year study was significantly higher than those of NSP group except NW (*p* < 0.05 to *p* < 0.01) (Table 4). The changes in RS, during 3‐year study period in the NSP group were negative and were significantly lower than those of SP group which were positive (*p* < 0.05 for day cough and wheeze and *p* < 0.01 for other cases), (Figure 2).

### 3.4 The effect of parental smoking on PFT

The PFT values were increased in NSP but decreased in SP asthmatic children the end of the study period, which were statistically significant for FEV1 and MEF50 in NSP group (*p* < 0.05 for both cases) (Table 5).

**TABLE 5:** Values of pulmonary function tests (PFT) and their percent change during 3 years in asthmatic children with non‐smoking (*n* = 58) and smoking parents (*n* = 32) at the beginning and at the end of the study

PFT	Non‐smokers				Smokers				*p* value end vs NS (% change)
	Beg	End	*p* value vs Beg	% change	Beg	End	*p* value vs Beg	% change	
FVC	71.1 ± 15.0	77.5 ± 15.2	NS	16.8 ± 36.4	65.9 ± 13.5	61.4 ± 12.1	NS	−2.6 ± 32.1	<0.05
FEV1	69.8 ± 15.2	77.9 ± 14.5	<0.05	19.6 ± 39.2	65.3 ± 12.8	60.7 ± 11.5	NS	−1.6 ± 35.1	<0.05
PEF	65.5 ± 16.5	71.9 ± 17.0	NS	20.2 ± 47.9	63.9 ± 14.6	60.1 ± 13.5	NS	1.5 ± 33.3	NS
MEF 50	62.5 ± 16.2	72.4 ± 19.2	<0.05	28.6 ± 61.3	59.4 ± 16.9	54.5 ± 15.8	NS	4.1 ± 86.1	NS

Values were present as mean ± SD. For comparison between the results of the beginning and the end of the study, paired *t* test and for comparison between two groups, unpaired *t*‐test were used.

Beg: beginning; NS: non‐smokers; FVC: forced vital capacity; FEV1: forced expiratory volume in 1 s; PEF: peak expiratory flow; MEF75, MEF50 and MEF25: maximal expiratory flow at 75%, 50% and 25% of the FVC, respectively. vs Beg: statistical comparison between the beginning and the end of 3 years of the study period, End vs NS: statistical comparison between asthmatic children with smoker parents with those on non‐smokers at the end of the study period.

The changes in PFT values during 3‐year study in the NSP group were positive, which were lower than the children of SP which were negative or very low, and the differences between the two groups were statistically significant for FVC and FEV1 (*p* < 0.05 for both cases) (Table 5).

## 4 DISCUSSION

Various types of drugs for the management of NSP group were decreased, which indicate the reduction of asthma severity during the 3‐year period with continues treatment, but the drugs were increased in SP children managed by the same physician indicating increased asthma severity caused by exposing to cigarette smoke of their parents.

The prevalence and severity of RS in the NSP group were also reduced during the 3‐year period, whilst in those with SP group both the prevalence and severity of RS were increased. The percent changes in the severity of RS during the study period in the SP patients were higher than those of NSP, which indicate intensifying of asthma severity by their parents smoking.

In the NSP group, PFT values were increased, but in those of SP, PFT values were reduced at the end of 3‐year study period. These objective results also support the intensifying of the asthma severity of asthmatic children exposing to cigarette smoke of their parents.

A strong link between parental smoking in the first 2 years of age and current parental smoking with the prevalence of wheeze, asthma and nocturnal cough was shown in a meta‐analysis^19^ and a systematic review.^20^ It was reported that among 10,314 children, more than 51% of them were exposed to environmental tobacco smoke (ETS) at home, and the prevalence of asthma symptoms was higher among ETS exposed children.^21^ The prevalence of ETS exposure at home in the students of high school in Kuwait was reported as 54%, and the prevalence of self‐reported asthma as 20.5%.^22^ Impaired lung function and increased risk of developing asthma in children with prenatal exposure to ETS were described by several studies.^11^, ^23^, ^24^

The above studies indicate the effect of parental smoking and ETS on the increasing prevalence of asthma among children and support the results of the present study. However, in the present study, the effect of parental smoking on severity of their asthmatic children was shown by the effects on RS, PFT values and drugs used for the management of the diseases during a 3‐year period that emphasised on the longitudinal studies for examining the effect of long‐term exposure of children to cigarette smoke on asthma.^25^

The effect of cigarette smoking on RS and PFT values was also shown in several studies including our previous studies,^26^, ^27^ and the effect of water pipe smoking on RS and PFT values was shown more pronounced than cigarette smoke even in smokers with deep inspiration during cigarette smoking,^28^ which support the effect of parents smoking on RS and PFT values of their asthmatic children. In the present study, the difference in the growth between asthmatic children with smoker and non‐smoker parents over study period (3 years) was not assessed, which should be examined in further studies.

## 5 CONCLUSION

In this longitudinal study, the effect of parental smoking on intensifying of their asthmatic children status was shown by increasing drug used for their management, prevalence and severity of RS but reducing their PFT values, indicating serious consequences of tobacco smoke exposure on asthmatic children by their parents smoking.

## CONFLICT OF INTEREST

The authors declare that they have no conflict of interest.

## ETHICS STATEMENT

**Right to privacy and informed consent**: The authors declare that no patient data appear in this article and have obtained the informed consent of patients and healthy people participated in this project.

**Protection of human subjects' research**: The authors declare that the procedures followed were in accordance with the regulations of the responsible Clinical Research Ethics Committee and in accordance with those of the World Medical Association and the Helsinki Declaration.

## AUTHOR CONTRIBUTIONS

Conception, designing of the work and final approval of the manuscript: MHB; acquisition and data analysis: AM, MB, AAH, MJ, AG and HA; and drafting the work or revising it critically for important intellectual content: AM and MHB.

## Data Availability

Data are available through corresponding authors upon request.
